# The effect of tranexamic acid on the risk of death and hysterectomy in women with post-partum haemorrhage: statistical analysis plan for the WOMAN trial

**DOI:** 10.1186/s13063-016-1332-2

**Published:** 2016-05-17

**Authors:** Haleema Shakur, Ian Roberts, Philip Edwards, Diana Elbourne, Zarko Alfirevic, Carine Ronsmans

**Affiliations:** Clinical Trials Unit, London School of Hygiene & Tropical Medicine, Keppel Street, London, WC1E 7HT UK; Division of Perinatal and Reproductive Medicine, University of Liverpool, Liverpool Women’s Hospital, Crown Street, Liverpool, L8 7SS UK; Infectious Diseases Epidemiology Unit, London School of Hygiene & Tropical Medicine, Keppel Street, London, WC1E 7HT UK

**Keywords:** Post-partum haemorrhage, Tranexamic acid, Clinical trial, Statistical analysis plan

## Abstract

**Background:**

Severe haemorrhage is a leading cause of maternal death worldwide. Most haemorrhage deaths occur soon after childbirth. Severe post-partum bleeding is sometimes managed by the surgical removal of the uterus (hysterectomy). Death and hysterectomy are important health consequences of post-partum haemorrhage, and clinical trials of interventions aimed at preventing these outcomes are needed.

**Methods:**

The World Maternal Antifibrinolytic trial aims to determine the effect of tranexamic acid on death, hysterectomy and other health outcomes in women with post-partum haemorrhage. It is an international, multicentre, randomised trial. Approximately 20,000 women with post-partum haemorrhage will be randomly allocated to receive an intravenous injection of either tranexamic acid or matching placebo in addition to usual care. The primary outcome measure is a composite of death in hospital or hysterectomy within 42 days of delivery. The cause of death will be described. Secondary outcomes include death, death due to bleeding, hysterectomy, thromboembolic events, blood transfusion, surgical and radiological interventions, complications, adverse events and quality of life. The health status and occurrence of thromboembolic events in breastfed babies will also be reported. We will conduct subgroup analyses for the primary outcome by time to treatment, type of delivery and cause of haemorrhage. We will conduct an analysis of treatment effect adjusted for baseline risk.

**Discussion:**

The World Maternal Antifibrinolytic trial should provide reliable evidence for the efficacy of tranexamic acid in the prevention of death, hysterectomy and other outcomes that are important to patients. We present a protocol update and the statistical analysis plan for the trial.

**Trial registration:**

Current Controlled Trials ISRCTN76912190 (Registration date 08 December 2008), Clinicaltrials.gov NCT00872469 (Registration date 30 March 2009) and Pan African Clinical Trials Registry: PACTR201007000192283 (Registration date 02 September 2010).

**Electronic supplementary material:**

The online version of this article (doi:10.1186/s13063-016-1332-2) contains supplementary material, which is available to authorized users.

## Introduction

Severe haemorrhage is associated with approximately one-quarter of maternal deaths worldwide [1]. Most of these deaths occur soon after childbirth. Other leading causes of maternal death are pre-existing medical conditions, high blood pressure and sepsis [2]. Severe post-partum bleeding is sometimes managed by the surgical removal of the uterus (hysterectomy). As well as the morbidity from the surgery, hysterectomy can cause psychological distress, particularly in women who want to have more children [3]. Death and hysterectomy are important health consequences of post-partum haemorrhage (PPH), and clinical trials of interventions aimed at preventing these outcomes are needed.

Tranexamic acid (TXA) reduces bleeding by inhibiting the enzymatic breakdown of the blood-clotting proteins fibrinogen and fibrin. A systematic review of clinical trials of TXA in surgery showed that TXA reduces blood loss by approximately one-third [4]. TXA also reduces mortality in bleeding trauma patients. When given soon after injury, TXA reduces the risk of death due to bleeding by approximately one-third [5, 6]. A randomised, controlled, open-label trial of TXA in women with PPH showed that TXA reduced blood loss but recommended that a larger international study is needed to investigate whether TA can decrease the need for invasive procedures and reduce maternal morbidity in women with PPH [7]. Also, a systematic review of TXA for the prevention and treatment of PPH concluded that insufficient evidence exists to support its use [8]. Taken together, reason exists to believe that TXA might reduce deaths due to bleeding following childbirth.

The World Maternal Antifibrinolytic (WOMAN) trial aims to determine the effect of TXA on death, hysterectomy and other health outcomes in women with post-partum haemorrhage. The trial protocol was published before the start of the trial [9]. The planned sample size was increased from 15,000 to 20,000 participants in May 2014. This paper gives the reason for this increase and presents the statistical analysis plan. Recruitment of 20,000 women should be complete by the summer of 2016. The statistical analysis plan was completed before the treatment allocation was un-blinded.

## Methods

### Study design and patients

The WOMAN trial is an international, multicentre, randomised, placebo-controlled trial. Adult women with post-partum haemorrhage following vaginal or caesarean section delivery who have a clinical diagnosis of post-partum haemorrhage are eligible for enrolment. The clinical diagnosis of PPH may be based on blood loss of over 500 mL after vaginal delivery or 1,000 mL after caesarean section or blood loss sufficient to compromise the haemodynamic stability. The fundamental eligibility criterion is the responsible clinician’s ‘uncertainty’ as to whether or not to use TXA in a particular woman with post-partum haemorrhage. Where TXA is indicated or was administered, or where it is contra-indicated, patients are not included in the trial. No pre-specified exclusion criteria exist. Patients receive all routine clinically indicated medical care and additionally are randomised to receive TXA or placebo. Hospitals where TXA is in routine use for PPH are not included in the trial. As the eligibility criteria automatically excluded patients who had received TXA, specific data on whether it was used before enrolment was not collected.

### Randomisation and masking

Randomisation codes were generated and secured by an independent statistical consultant from Sealed Envelope Ltd (London, UK). Trial treatment packs are prepared in accordance with the randomisation list. Women eligible for inclusion are randomised to receive either active (TXA) or placebo (sodium chloride 0.9 %) intravenously. Baseline information is collected on the trial entry form, and the next lowest consecutively numbered pack is taken from a box of eight treatment packs. Once the treatment ampoule is confirmed as intact, the patient is considered to be randomised. Once a patient has been randomised, the outcome for the woman is obtained even if the trial treatment is interrupted or not given. Both participants and study staff (site investigators and trial coordinating centre staff) are masked to treatment allocation. An emergency un-blinding service is available.

### Trial procedures

After eligibility is confirmed and the appropriate consent procedure followed, each patient is assigned a uniquely numbered treatment pack. In this way they are randomly allocated to receive 1 gram of TXA or placebo by intravenous injection. If bleeding continues after 30 minutes, or if bleeding stops and restarts within 24 h, a second dose of 1 g of TXA or placebo may be given. No pre-established maximum time between the start of PPH and administration of the study treatment exists. After a patient has been randomised, the outcome in hospital is collected even if the trial treatment is interrupted or is not actually given. Outcome is collected at 6 weeks (42 days, as this usually defines the post-partum period) after randomisation, at discharge from the randomising hospital or at death (whichever occurs first). Adverse events are reported in-hospital and after discharge up to day 42.

### Ethics approval

Before the trial can start at a site, all relevant regulatory and ethics approvals are obtained. An additional file presents the current list of ethics approval for participating countries and sites (see Additional file 1).

### Consent

The trial is being conducted in accordance with the International Conference on Harmonisation of Technical Requirements for Registration of Pharmaceuticals for Human Use–Good Clinical Practice (ICH-GCP), and participant consent is obtained in accordance with the approved protocol. The consent procedure used in the WOMAN Trial is detailed in the published protocol [9]. The informed consent procedure used at each site is approved by the relevant ethics and regulatory agencies. In summary, consent is obtained from patients if physical and mental capacity allows consent. If a patient cannot give consent, proxy consent is obtained from a relative or representative. If a proxy was unavailable, then if permitted by local regulation, consent is deferred or waived. When consent is deferred or given by a proxy, the patient is informed about the trial as soon as possible, and consent obtained for use of the data collected, if needed.

## Sample size

Before the trial started, we anticipated a baseline event rate of 2.5 % for death and 2.5 % for hysterectomy. Assuming a control group event rate of 2.5 % for death and 2.5 % for hysterectomy and that 1 % of women die after hysterectomy, a study with 15,000 women should have more than 90 % power to detect a 25 % reduction from 4 % to 3 % in the primary endpoint of death or hysterectomy at the 5 % significance level. Expected loss to follow-up (less than 1 %) should not impact importantly on the power (at the time of writing this Statistical Analysis Plan, we had achieved 99 % follow-up at 42 days after randomisation, at discharge from hospital or death).

However, because the case fatality observed in the trial (by December 2013) was higher (3 %) than expected, a modest increase in the sample size from 15,000 to 20,000 women would mean that the trial may have sufficient (90 %) power to detect a 25 % reduction in maternal mortality alone at the 5 % significance level. This increase also provided additional power to detect an effect on the composite endpoint of death or hysterectomy, whilst providing protection against the possibility that the effect of TXA on death and hysterectomy is different. Increasing the sample size to 20,000 women involved an extra 15 months of recruitment.

The primary outcome remains the same (i.e. a composite of death and hysterectomy) with death alone as a secondary outcome. The independent Data Monitoring Committee was aware of the higher case fatality, as these data were presented as part of their routine review. The independent Trial Steering Committee also reviewed and approved the sample size increase.

## Statistical analysis

### Trial profile

**Fig. 1:**
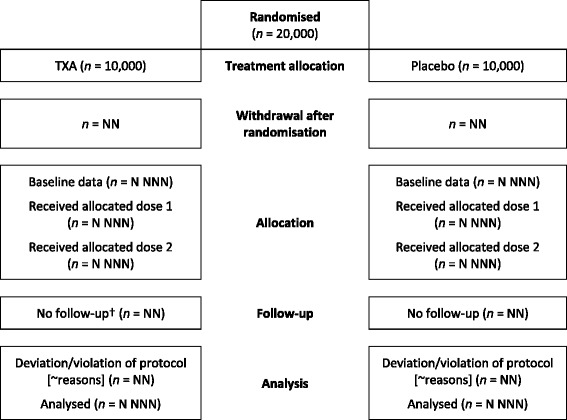
Trial profile. ^†^No follow-up relates to those patients where there is no information on the primary endpoint

The flow of study participants will be displayed in a CONSORT diagram [10], as shown in Fig. 1. Because the trial involves a life-threatening emergency situation, we did not require clinicians to complete a screening log. The number of patients included in the primary and secondary analyses, the reasons for exclusions and the number of patients lost to follow-up will be reported.

Patients randomised who did not fulfil the eligibility criteria and those who did not receive their allocated treatment will be considered as having deviated from the protocol. Their data will be included in the intention-to-treat (ITT) analysis. If a patient or their representative withdraws consent for data collection, only data up to the point of withdrawal will be used in the analysis. Additionally, we will summarise the type of consent process used (number and percentage): patient, personal representative, professional representative and waiver.

## Planned analysis

### Primary analysis

The main analyses will compare all those allocated tranexamic acid versus those allocated placebo, on an ‘intention-to-treat’ basis, irrespective of whether they received the allocated treatment or not.

### Baseline characteristics

To check that randomisation produced broadly similar groups, the baseline characteristics obtained prior to randomisation will be presented as the number and percentage in each group (Table 1).

**Table 1:** Baseline characteristics of participants prior to randomisation

	Tranexamic acid	Placebo
	(*n* = 10,000)	(*n* = 10,000)
Age at randomisation (years):		
< 16	N (X %)	N (X %)
16–25	N NNN (X %)	N NNN (X %)
26–33	N NNN (X %)	N NNN (X %)
34+	N NNN (X %)	N NNN (X %)
Baby delivered in this hospital	N NNN (X %)	N NNN (X %)
Type of delivery:		
Vaginal	N NNN (X %)	N NNN (X %)
Caesarean section	N NNN (X %)	N NNN (X %)
Time between delivery and randomisation (hours):		
≤ 1	N NNN (X %)	N NNN (X %)
> 1 to ≤ 3	N NNN (X %)	N NNN (X %)
> 3^a^	N NNN (X %)	N NNN (X %)
Placenta fully delivered:		
Yes	N NNN (X %)	N NNN (X %)
No	NNN (X %)	NNN (X %)
Primary cause of haemorrhage:		
Uterine atony	N NNN (X %)	N NNN (X %)
Placenta praevia/accreta	N NNN (X %)	N NNN (X %)
Surgical trauma/tears	N NNN (X %)	N NNN (X %)
Other	N NNN (X %)	N NNN (X %)
Unknown	N NNN (X %)	N NNN (X %)
Systolic BP (mmHg):		
≥ 90	N NNN (X %)	N NNN (X %)
< 90	N NNN (X %)	N NNN (X %)
Estimated volume of blood lost (mL):		
≤ 500	N NNN (X %)	N NNN (X %)
> 500 to ≤ 1000	N NNN (X %)	N NNN (X %)
> 1000	N NNN (X %)	N NNN (X %)
Uterotonic prophylaxis given:		
Yes	N NNN (X %)	N NNN (X %)
No	N NNN (X %)	N NNN (X %)
Not known	N NNN (X %)	N NNN (X %)
Clinical signs of haemodynamic instability:		
Yes	N NNN (X %)	N NNN (X %)
No	N NNN (X %)	N NNN (X %)

^a^Includes X patients randomly assigned more than 24 h after delivery

### Primary outcome: death or hysterectomy

The primary outcome is a composite of death or hysterectomy. Death and hysterectomy are important outcomes for women, are easily measured and could potentially be prevented by TXA. We use this composite endpoint to increase the number of outcome events, thereby increasing the statistical power to detect a treatment effect [11]. Although the increase in power comes at a cost of greater uncertainty in interpretation of the results, the use of a composite endpoint is justified if the components are of similar importance, occur with similar frequency and are likely to be affected to a similar degree by the treatment [12, 13]. Whilst hysterectomy cannot be considered as important as death, the composite may be justified on the basis that hysterectomy is conducted as a last resort to prevent death from bleeding, in which case this less serious outcome may be a reasonable surrogate for maternal death.

We anticipated that death and hysterectomy would occur with similar frequency. Whether death and hysterectomy will be affected to a similar degree by TXA is unknown, but to the extent that death and hysterectomy are direct consequences of bleeding, we should expect effects in the same direction, if not of a similar magnitude.

During the trial, site monitoring showed that some hysterectomies are conducted before randomisation. For example, in response to the onset of severe life-threatening bleeding during caesarean section, an obstetrician may decide to conduct a hysterectomy, and while the hysterectomy is underway, the woman is enrolled into the trial. Similarly, in some cases of uterine rupture, a hysterectomy is sometimes done prior to randomisation. In other cases, a hysterectomy was conducted in a peripheral hospital, and the woman is enrolled into the WOMAN trial after having been transferred for the management of ongoing bleeding. Although TXA might reduce the risk of death in these cases, it could not affect the risk of hysterectomy because the hysterectomy was carried out prior to randomisation. Including such patients in the analysis will dilute the treatment effect towards the null. Therefore, only hysterectomies carried out after randomisation will be included. All hysterectomies will be monitored before database lock using the Primary Outcome Monitoring Form to confirm which ones were done after randomisation (see Additional file 2).

Pearson’s chi-squared test will be used as the primary test of statistical significance of the effect of treatment allocation on the composite of death or hysterectomy by 42 days. Frequencies and percentages per arm, and the risk ratio for the outcome with the treatment with its 95 % confidence interval (CI) will be reported as shown in Table 2.

**Table 2:** Effect of tranexamic acid (TXA) on death or hysterectomy

	TXA	Placebo	Risk ratio (95 % CI)	*p* value*
	(*n* = 10,000)	(*n* = 10,000)		
Death or hysterectomy	NN (X %)	NN (X %)	X.XX (X.XX–X.XX)	*p* = 0.XX
Death (all causes)	NN (X %)	NN (X %)	X.XX (X.XX–X.XX)	*p* = 0.XX
Bleeding deaths	NN (X %)	NN (X %)	X.XX (X.XX–X.XX)	*p* = 0.XX
Disseminated intravascular coagulation deaths	NN (X %)	NN (X %)	X.XX (X.XX–X.XX)	*p* = 0.XX
Pulmonary embolic deaths	NN (X %)	NN (X %)	X.XX (X.XX–X.XX)	*p* = 0.XX
Sepsis deaths	NN (X %)	NN (X %)	X.XX (X.XX–X.XX)	*p* = 0.XX
Deaths due to other causes	NN (X %)	NN (X %)	X.XX (X.XX–X.XX)	*p* = 0.XX
Hysterectomy	NN (X %)	NN (X %)	X.XX (X.XX–X.XX)	*p* = 0.XX

*p values from Pearson’s chi-squared test

### Imputation of missing data

As loss to follow-up is expected to be minimal (i.e. less than 1 % missing data on the primary outcome), missing values will not be imputed.

### Subgroup analyses

All subgroups will be defined according to variables that are measured before randomisation and will be presented as shown in Table 3. The following subgroup analyses will be carried out for the primary outcome (death or hysterectomy), unless otherwise stated.

**Table 3:** Effect of tranexamic acid (TXA) on death or hysterectomy by subgroups

Subgroup	TXA		Placebo		Risk ratio (99 % CI)
	Death or hysterectomy	Received TXA	Death or hysterectomy	Received Placebo	
Time between delivery and randomisation:					
≤ 1 h	NN (X %)	N NNN	NN (X %)	N NNN	X.XX (X.XX–X.XX)
1–3 h	NN (X %)	N NNN	NN (X %)	N NNN	X.XX (X.XX–X.XX)
> 3 h	NN (X %)	N NNN	NN (X %)	N NNN	X.XX (X.XX–X.XX)
(p = 0.XXX)*					
Type of delivery:					
Vaginal	NN (X %)	N NNN	NN (X %)	N NNN	X.XX (X.XX–X.XX)
Caesarean section	NN (X %)	N NNN	NN (X %)	N NNN	X.XX (X.XX–X.XX)
* (p = 0.XXX)*					
Primary cause of haemorrhage:					
Uterine atony	NN (X %)	N NNN	NN (X %)	N NNN	X.XX (X.XX–X.XX)
Other/unknown	NN (X %)	N NNN	NN (X %)	N NNN	X.XX (X.XX–X.XX)
* (p =* 0.XXX*)*					

Notes

*p values from unadjusted tests of interaction in a logistic regression model to assess evidence for whether the effect of treatment differs across subgroup categories

#### Time to treatment

The WOMAN trial was planned before the results of the CRASH-2 trial of TXA in bleeding trauma patients were available. The CRASH-2 trial, which recruited 20,210 adults with significant traumatic bleeding, showed that TXA reduces death due to bleeding (RR = 0.85, 95 % CI 0.76–0.96) and all-cause mortality (RR = 0.91, 95 % CI 0.85–0.97), with no increase in vascular occlusive events. The CRASH-2 trial investigators hypothesised that TXA would be most effective when given soon after injury, when bleeding is profuse, but less effective later, when the acute phase response to trauma increases the risk of thrombosis. They tested this hypothesis by conducting a pre-specified subgroup analysis of the effect of TXA by time from injury to the start of treatment (≤ 1, 1– ≤ 3, 3–8 h) and found strong evidence in support of their hypothesis (test of homogeneity *p* < 0.0001). In patients treated within 3 h of injury, TXA substantially reduced the risk of death due to bleeding, but when given after 3 h, TXA appeared to increase the risk of death due to bleeding [6].

Early activation of fibrinolysis is common after trauma and is associated with increased mortality [14]. Tissue plasminogen activator (TPA), the enzyme that converts plasminogen to the active fibrinolytic enzyme plasmin, is stored within the vascular endothelium in secretory organelles called Weibel Palade bodies. Trauma triggers the rapid release of tissue plasminogen activator (TPA) from the vascular endothelium resulting in fibrinolysis and increased bleeding [15]. By inhibiting early fibrinolysis, TXA reduces the risk of exsanguination. The apparent increase in death due to bleeding with late TXA administration may reflect PAI-1-induced suppression of fibrinolysis and the onset of thrombotic disseminated intravascular coagulation (DIC) [16]. The latter is characterised by intravascular activation of coagulation with widespread fibrin deposition. By inhibiting fibrinolysis, TXA could worsen thrombotic DIC. Although the underlying pathology is thrombotic, due to the consumption of coagulation factors, thrombotic DIC usually manifests as bleeding.

Similar temporal changes in fibrinolysis have been observed after childbirth [17]. Within 1 h of delivery, the serum concentration of TPA doubles, possibly due to the trauma of childbirth. Thereafter, the TPA concentration falls steeply. On the other hand, levels of the plasminogen activator inhibitors (PAI-1 and PAI-2) are increased around the time of delivery and remain so for several days. We might therefore expect TXA would be most effective when given soon after delivery, when TPA levels are highest, and less effective (possibly even harmful) when given several hours after delivery, when the risk of thrombotic DIC may be increased. We will examine this hypothesis by conducting a sub-group analysis of the effect of TXA according to the time interval between delivery and TXA treatment (≤1, > 1 to ≤ 3, > 3 h). The outcome measure for this subgroup analysis will be death due to bleeding. The results of this analysis will be considered in the context of the relevant biological and clinical data on the time to treatment interaction.

#### Type of delivery

Because a substantial proportion of all deliveries are by caesarean section and caesarean section is an established risk factor for post-partum haemorrhage [18], it is important to examine whether the effect of TXA on the risk of death and hysterectomy varies by type of delivery. We do not anticipate substantial heterogeneity by type of delivery.

#### Primary cause of post-partum haemorrhage

For post-partum haemorrhage due to uterine atony, uterotonics are recommended as the first line treatment [19]. It is believed that uterine contraction will compress the spiral arteries and reduce bleeding. When planning the WOMAN trial, some reviewers expressed concern that administration of the WOMAN trial treatment might lead to fewer women receiving prophylactic uterotonics. For this reason, we collected information on uterotonic use and planned a sub-group analysis on the basis of whether or not uterotonics were administered. It was suggested that TXA would be less effective in women who receive uterotonics, particularly when uterine atony is the cause of bleeding. However, because 99 % of women recruited to May 2014 received uterotonics, the planned subgroup analysis would be uninformative. Instead, we will examine the effect of TXA by cause of the bleeding (uterine atony versus all other causes) as determined at baseline. We do not anticipate substantial heterogeneity by cause of haemorrhage.

The main analysis for the pre-specified subgroups will be an unadjusted test of interaction in a logistic regression model to assess evidence for whether the effect of treatment differs across subgroup categories. Unless there is strong evidence against the null hypothesis of homogeneity of effects (i.e. *p* < 0.001), the overall relative risk will be considered the most reliable guide to the approximate relative risks in all subgroups. For each subgroup, we will report frequencies and percentages per arm with the RR and 99 % CI as estimates of stratum-specific treatment effects, with the *p* value for the test of interaction.

### Secondary outcomes

#### Death

**Fig. 2:**
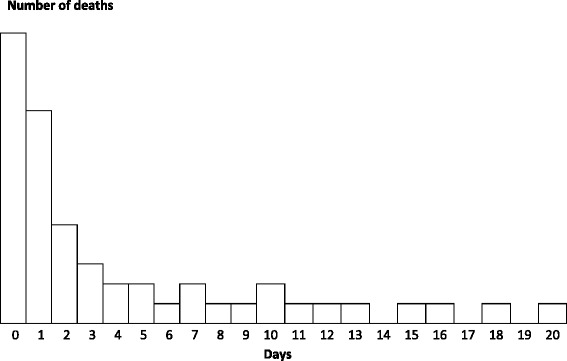
Distribution of cause of death by days since randomisation. Notes: Bars will be stacked to show number of deaths due to bleeding, pulmonary embolism, or other causes.

Death will be analysed (separately from hysterectomy) as a secondary outcome. As outlined above, although the use of a composite endpoint increases the statistical power, if the effect of TXA on death and hysterectomy are not similar, it could make interpretation of the primary results difficult. The causes of death will be analysed as shown in Table 2. Also, there is no reason to believe that TXA can prevent deaths from causes such as eclampsia or sepsis, unless death from these other causes is made more likely by bleeding. Therefore, we will assess the effect of TXA on all-cause mortality and cause-specific mortality (death due to bleeding, pulmonary embolism and other causes (Table 2). We will present the distribution of causes of death by days since randomisation as described in Fig. 2.

#### Thromboembolic events

An increased risk of venous thromboembolic events (VTE) persists for several weeks post-partum [20]. Venous thrombosis is one of the leading causes of maternal morbidity and mortality with about 95 % of these events occurring during the first 4 weeks post-partum [21]. The risk of deep vein thrombosis (DVT) is believed to be higher than pulmonary embolism (PE) in the post-partum period. Any further increase in risk from TXA use would be important. We will therefore assess and present the risk of venous (DVT and PE) and arterial events (myocardial infarction (MI) and stroke) as shown in Table 4. The definitions of DVT, PE, stroke and MI used in the trial are shown in Additional file 3.

**Table 4:** Effect of tranexamic acid (TXA) on thromboembolic events, need for surgery and transfusion and level of dependency

	TXA	Placebo	Risk ratio (95 % CI)	*p* value*
	(*n* = 10,000)	(*n* = 10,000)		
Thromboembolic events				
Any event	NN (X %)	NN (X %)	X.XX (X.XX–X.XX)	*p* = 0.XX
Venous events (DVT, PE)	NN (X %)	NN (X %)	X.XX (X.XX–X.XX)	*p* = 0.XX
DVT	NN (X %)	NN (X %)	X.XX (X.XX–X.XX)	*p* = 0.XX
PE	NN (X %)	NN (X %)	X.XX (X.XX–X.XX)	*p* = 0.XX
Arterial events (MI, stroke)	NN (X %)	NN (X %)	X.XX (X.XX–X.XX)	*p* = 0.XX
MI	NN (X %)	NN (X %)	X.XX (X.XX–X.XX)	*p* = 0.XX
Stroke	NN (X %)	NN (X %)	X.XX (X.XX–X.XX)	*p* = 0.XX
Surgical interventions				
Hysterectomy	NN (X %)	NN (X %)	X.XX (X.XX–X.XX)	*p* = 0.XX
Manual removal of placenta	NN (X %)	NN (X %)	X.XX (X.XX–X.XX)	*p* = 0.XX
Intrauterine tamponade	NN (X %)	NN (X %)	X.XX (X.XX–X.XX)	*p* = 0.XX
Embolisation	NN (X %)	NN (X %)	X.XX (X.XX–X.XX)	*p* = 0.XX
Brace sutures of uterus	NN (X %)	NN (X %)	X.XX (X.XX–X.XX)	*p* = 0.XX
Arterial ligation	NN (X %)	NN (X %)	X.XX (X.XX–X.XX)	*p* = 0.XX
Laparotomy	NN (X %)	NN (X %)	X.XX (X.XX–X.XX)	*p* = 0.XX
Any surgical or radiological intervention	NN (X %)	NN (X %)	X.XX (X.XX–X.XX)	*p* = 0.XX
Blood transfusion				
Whole blood	NN (X %)	NN (X %)	X.XX (X.XX–X.XX)	*p* = 0.XX
Packed red blood cells	NN (X %)	NN (X %)	X.XX (X.XX–X.XX)	*p* = 0.XX
Frozen plasma	NN (X %)	NN (X %)	X.XX (X.XX–X.XX)	*p* = 0.XX
Other blood products	NN (X %)	NN (X %)	X.XX (X.XX–X.XX)	*p* = 0.XX
Any transfusion	NN (X %)	NN (X %)	X.XX (X.XX–X.XX)	*p* = 0.XX
Blood transfusion (units)				
Whole blood	mean (SD)	mean (SD)	Diff in means (X.X – X.X)	*p* = 0.XX
Packed red blood cells	mean (SD)	mean (SD)	Diff in means (X.X – X.X)	*p* = 0.XX
Frozen plasma	mean (SD)	mean (SD)	Diff in means (X.X – X.X)	*p* = 0.XX
Other blood products	mean (SD)	mean (SD)	Diff in means (X.X – X.X)	*p* = 0.XX
Any transfusion	mean (SD)	mean (SD)	Diff in means (X.X – X.X)	*p* = 0.XX
EQ-5D^a^				
Mobility	NN (X %)	NN (X %)	X.XX (X.XX–X.XX)	*p* = 0.XX
Self-care	NN (X %)	NN (X %)	X.XX (X.XX–X.XX)	*p* = 0.XX
Usual activities	NN (X %)	NN (X %)	X.XX (X.XX–X.XX)	*p* = 0.XX
Pain/discomfort	NN (X %)	NN (X %)	X.XX (X.XX–X.XX)	*p* = 0.XX
Anxiety/depression	NN (X %)	NN (X %)	X.XX (X.XX–X.XX)	*p* = 0.XX
Visual analogue scale	mean (SD)	mean (SD)	Diff in means (X.X – X.X)	*p* = 0.XX

*DVT* deep vein thrombosis, *MI* myocardial infarction, *PE* pulmonary embolism

*p values from Pearson’s chi-squared test

^a^Proportion with severe problems

#### Surgical and radiological interventions to treat haemorrhage

Hysterectomy will be analysed as a secondary outcome (Table 2). Additionally, the following interventions will be analysed: manual removal of the placenta, intrauterine tamponade, embolisation, brace sutures of the uterus, arterial ligation and laparotomy for other reasons (carried out after randomisation to control bleeding and achieve haemostasis for procedures other than those listed). If TXA reduces bleeding, it may also reduce the need for these additional treatments. However, as the decision to randomise can be made at the same time, or even after these interventions are carried out, some dilution of the treatment effect towards the null might be expected. Surgical and radiological events will be analysed in two ways: as individual events and grouped as ‘surgical and radiological events’. These will be summarised as the number and percentage of patients per arm. These will be presented as shown in Table 4.

#### Blood transfusion

TXA has been shown to reduce the probability of receiving a blood transfusion by one-third in patients undergoing routine surgery [4]. If TXA can reduce the need for blood product transfusion in women with PPH, this would be an important finding. We will therefore assess the effect of TXA on the use of whole blood or packed red blood cells, fresh frozen plasma and other blood products in transfusion. These will be summarised as the number and percentage of patients per arm who receive these, and the mean, (SD) or median (range) as appropriate, units transfused per treatment arm. The effect of treatment allocation will be tested using a two-sample *t*-test (or Mann-Whitney rank sum test, as appropriate). However, once again, since the decision to transfuse is often made before or at the time of randomisation, some dilution towards the null is to be expected. The results will be presented as shown in Table 4.

#### Quality of life

The impact on the quality of life of women who suffer PPH is not well researched. One study suggests that the health and quality of life of women after maternal morbidities such as PPH, negatively affects the woman’s wellbeing and quality of life [22]. Information on the health status of women is collected on the outcome form using the EQ-5D. This consists of two principal measurement components. The first defines health-related quality of life in terms of five dimensions: ‘mobility’, ‘self-care’, ‘usual activities’, ‘pain/discomfort’ and ‘anxiety/depression’ [23]. Responses in each dimension are divided into three ordinal levels, coded: (1) no problems, (2) some or moderate problems and (3) severe or extreme problems. The second measurement component of the EQ-5D consists of a 20-cm vertical visual analogue scale ranging from 100 (best imaginable health state) to 0 (worst imaginable health state), which provides an indication of the woman’s health status. EQ-5D Scores will be presented as the proportions of women reporting problems (i.e. some, moderate, severe or extreme) on each EQ-5D dimension. In addition, the means (with SD) of the EQ-5D visual analogue scales per arm and the difference in the means will also be reported in order to assess the treatment effect, along with the *p* value from a two-sample *t*-test. These will be presented as shown in Table 4.

#### Complications

Women with PPH are at risk of other significant medical events associated with pregnancy and PPH. These include renal failure, cardiac failure, respiratory failure, hepatic failure, sepsis and seizure, and these events are collected routinely as outcomes. An increase in the risk of seizure using high doses of TXA has been reported [24, 25]. These will be presented as shown in Table 5.

**Table 5:** Complications

Type of adverse event	Tranexamic acid	Placebo	Risk ratio (95 % CI)	*p* value*
	(n = 10,000)	(n = 10,000)		
Renal failure	N (X %)	N (X %)	X.XX (X.XX–X.XX)	*p* = 0.XX
Cardiac failure	N (X %)	N (X %)	X.XX (X.XX–X.XX)	*p* = 0.XX
Respiratory failure	N (X %)	N (X %)	X.XX (X.XX–X.XX)	*p* = 0.XX
Hepatic failure	N (X %)	N (X %)	X.XX (X.XX–X.XX)	*p* = 0.XX
Sepsis	N (X %)	N (X %)	X.XX (X.XX–X.XX)	*p* = 0.XX
Seizure	N (X %)	N (X %)	X.XX (X.XX–X.XX)	*p* = 0.XX

Notes

*p values from Pearson’s chi-squared test

#### Adverse events

Other untoward medical events are collected up to 42 days after randomisation as adverse events (AEs). In line with ICH-GCP guidelines [26], an AE is considered as serious if it results in death, is life-threatening, requires inpatient hospitalisation or prolongation of existing hospitalisation, results in persistent or significant disability/incapacity or results in a congenital anomaly/birth defect. Where there is at least a possibility of an adverse event being causally linked to the trial drug, this is considered to be an adverse reaction. A suspected unexpected serious adverse reaction (SUSAR) is an unexpected occurrence of a serious adverse reaction. There need only to be an index of suspicion that the event is a previously unreported reaction to a trial drug or a previously reported but exaggerated or unexpectedly frequent adverse drug reaction.

The number of AEs, SAEs, and SUSARs grouped by MedDRA® codes [27] and the number of patients with at least one event will be compared between arms using a chi-squared test (or Fisher’s exact test), with RRs and 95 % CI when these are computable. Frequencies and percentages per treatment group will be presented as shown in Table 6.

**Table 6:** Adverse events

Type of adverse event^a^	Tranexamic acid	Placebo	Risk ratio (95 % CI)	*p* value*
	(*n* = 10,000)	(*n* = 10,000)		
MedDRA code groups	N (X %)	N (X %)	X.XX (X.XX–X.XX)	*p* = 0.XX

Notes

*p values from Pearson’s chi-squared test (or Fisher’s exact test)

^a^AE’s, SAE, and SUSAR grouped by MedDRA® codes

#### Status of babies

TXA is believed to pass into breast milk to a concentration of approximately one hundredth of the concentration in the maternal blood [28]. The number and percentage of babies born alive per treatment group will be described. Where babies were born alive and breastfed during the course of the follow-up period, their status (alive or dead) will be presented as shown in Table 7.

**Table 7:** Death or thromboembolic events in breast-fed babies^a^

	Tranexamic acid	Placebo	Risk ratio (95 % CI)	*p* value*
	(*n* = 10,000)	(*n* = 10,000)		
Any death (of breastfed baby)	NN (X %)	NN (X %)	X.XX (X.XX–X.XX)	*p* = 0.XX
Any thromboembolic event (of breastfed baby)	NN (X %)	NN (X %)	X.XX (X.XX–X.XX)	*p* = 0.XX

*p values from Pearson’s chi-squared test

^a^Babies born alive and breastfed for some duration

#### Thromboembolic events in breastfed babies

Data on any thromboembolic events (TE) in babies are collected. TE was defined as follows: the event must be a confirmed diagnosis and may include any venous or arterial thrombosis (thrombosis of limb artery/deep veins, renal artery/veins, pulmonary embolism, hepatic veins, caval veins, intracardiac thrombosis, portal vein, mesenteric veins/artery, cerebral veins, retinal vein, ischemic stroke, arteries, aorta, myocardial infarction, microvascular thrombosis from purpura fulminans or disseminated intravascular coagulation. These data will be presented as shown in Table 7.

For all events reported in Tables 4, 5, 6 and 7, frequencies and percentages per arm will be presented, with a RR and 95 % CI as estimates of treatment effect, along with the *p* value from Pearson’s chi-squared test.

## Other analyses: to be reported in a separate publication

### Analysis 1: Adjusting for possible imbalance in baseline prognostic factors

In a large trial such as the WOMAN trial, baseline characteristics of patients that may influence the outcome should be evenly distributed between the treatment and placebo groups, so that any difference in outcome can be attributed to the intervention. However, it is still possible that a chance imbalance in important prognostic factors could influence the results [29, 30]. To investigate this possibility, we will conduct an analysis of the effect of treatment that is adjusted for baseline risk. We will build a prognostic model based on pre-specified baseline variables and use it to estimate the predicted risk of the outcome at baseline.

The primary outcome is death or hysterectomy; death due to bleeding is an important secondary outcome. The most important prognostic factors for these outcomes that are measured at baseline are maternal age, estimated blood loss, systolic blood pressure and haemodynamic instability. These variables will be included in a multivariable prognostic model based on the final trial dataset. Although there are almost complete data on these variables, in the case of missing data, the missing values will be replaced by the mean of the observed data [31].

The trial data will then be stratified into risk deciles as shown in Tables 8 and 9 based on the predicted risk of the outcomes at baseline. We will report frequencies and percentages within each risk decile, and calculate a risk ratio (with 95 % CI) for each risk decile. The pooled risk ratio (with a 95 % CI) will be estimated as an inverse variance weighted average of the stratum-specific risk ratios. The pooled risk ratio should provide an estimate of the treatment effect that is un-confounded by baseline risk. The advantage of this approach is that the effect of baseline risk on the treatment effect is more explicit than when covariate-adjusted odds ratios are calculated using logistic regression. Furthermore, risk ratios are easier to interpret and apply to individual patients than are odds ratios [32].

**Table 8:** Death or hysterectomy by predicted risk at baseline

Risk decile^a^	Tranexamic acid (TXA)		Placebo		Risk ratio (95 % CI)
	Death or hysterectomy	Received TXA	Death or hysterectomy	Received placebo	
1	NN (X %)	N NNN	NN (X %)	N NNN	X.XX (X.XX–X.XX)
2	NN (X %)	N NNN	NN (X %)	N NNN	X.XX (X.XX–X.XX)
3	NN (X %)	N NNN	NN (X %)	N NNN	X.XX (X.XX–X.XX)
4	NN (X %)	N NNN	NN (X %)	N NNN	X.XX (X.XX–X.XX)
5	NN (X %)	N NNN	NN (X %)	N NNN	X.XX (X.XX–X.XX)
6	NN (X %)	N NNN	NN (X %)	N NNN	X.XX (X.XX–X.XX)
7	NN (X %)	N NNN	NN (X %)	N NNN	X.XX (X.XX–X.XX)
8	NN (X %)	N NNN	NN (X %)	N NNN	X.XX (X.XX–X.XX)
9	NN (X %)	N NNN	NN (X %)	N NNN	X.XX (X.XX–X.XX)
10	NN (X %)	N NNN	NN (X %)	N NNN	X.XX (X.XX–X.XX)
Overall	NN (X %)	N NNN	NN (X %)	N NNN	X.XX (X.XX–X.XX)
(*I* ^*2*^ = X %, *p* = 0.XX)					

Notes

^a^Deciles of the predicted risk of the outcome at baseline, using a prognostic model including the baseline variables maternal age, estimated blood loss, systolic blood pressure and haemodynamic instability

**Table 9:** Death due to bleeding by predicted risk at baseline

Risk decile^a^	Tranexamic acid (TXA)		Placebo		Risk ratio (95 % CI)
	Death due to bleeding	Received TXA	Death due to bleeding	Received placebo	
1	NN (X %)	N NNN	NN (X %)	N NNN	X.XX (X.XX–X.XX)
2	NN (X %)	N NNN	NN (X %)	N NNN	X.XX (X.XX–X.XX)
3	NN (X %)	N NNN	NN (X %)	N NNN	X.XX (X.XX–X.XX)
4	NN (X %)	N NNN	NN (X %)	N NNN	X.XX (X.XX–X.XX)
5	NN (X %)	N NNN	NN (X %)	N NNN	X.XX (X.XX–X.XX)
6	NN (X %)	N NNN	NN (X %)	N NNN	X.XX (X.XX–X.XX)
7	NN (X %)	N NNN	NN (X %)	N NNN	X.XX (X.XX–X.XX)
8	NN (X %)	N NNN	NN (X %)	N NNN	X.XX (X.XX–X.XX)
9	NN (X %)	N NNN	NN (X %)	N NNN	X.XX (X.XX–X.XX)
10	NN (X %)	N NNN	NN (X %)	N NNN	X.XX (X.XX–X.XX)
Overall	NN (X %)	N NNN	NN (X %)	N NNN	X.XX (X.XX–X.XX)
(*I* ^*2*^ = X %, *p* = 0.XX)					

Notes

^a^Deciles of the predicted risk of the outcome at baseline, using a prognostic model including the baseline variables maternal age, estimated blood loss, systolic blood pressure and haemodynamic instability

A forest plot will be prepared to show graphically how the treatment effect varies by baseline risk. We will use a chi-squared test to assess any heterogeneity in the treatment effect across the risk groups, and we will calculate the I-squared statistic to quantify the percentage of variability in effect estimates that is due to heterogeneity rather than chance. To reduce the likelihood of making inappropriate inferences, we pre-specify that unless there is strong evidence against the null hypothesis of homogeneity of effects (i.e. *p* < 0.001), the pooled relative risk will be considered the most reliable guide to the approximate treatment effects in all risk strata. We do not anticipate substantial heterogeneity by baseline risk. However, we hypothesise that the risk reduction with TXA would be greatest in patients at low risk of death since a smaller proportion of these women will have un-survivable bleeding.

### Analysis 2: Cost-effectiveness

An economic analysis will be relevant if TXA clearly demonstrates efficacy in achieving its clinical aims. In this case, the study will be undertaken in the form of a cost-effectiveness analysis with the aim of estimating the incremental cost-effectiveness ratio comparing the use of TXA with normal clinical practice. Analysis will be based on adjusted life years gained. A further analysis will explore the use of the EQ-5D data to quality-adjusted survival. In this study, the economic analysis is clearly bounded as virtually all significant resource use will occur in the initial period of hospitalisation. As such, neither a long-term resource analysis nor an analysis of out-of-hospital costs will be required. The trial use of TXA is likely to mirror its use in normal clinical practice, hence the cost-effectiveness estimated in the trial (adjusted for protocol driven costs) will closely approximate cost-effectiveness in actual clinical practice. Data on physical resource consumption (e.g. the length and nature of hospital stay) will be collected for each patient and a common unit cost at a country level will be applied. A sensitivity analysis will be undertaken to assess the robustness of the economic analysis in response to variations in key variables such as drug prices. In all cases, the economic analysis will be integrated with the clinical trial procedures to optimise efficiency and minimise inconvenience to patients.

Mechanical ventilation and intensive care unit (ICU) admission are resource consumption measures and are not required for all patients. Mechanical ventilation will be summarised as number and percentage of patients per arm who received such a therapy. Where mechanical ventilation is used exclusively for general anaesthetic for surgery, this data is excluded. Length of stay in the ICU and the hospital will be censored due to early deaths or by a stay in the ICU or hospital longer than 42 days. Summary statistics will include the median and the interquartile range computed separately for each treatment arm.

## Data monitoring and interim analyses

An independent Data Monitoring Committee (DMC) is responsible for reviewing the progress of the trial, including recruitment, data quality and main outcomes and safety data. The DMC has the responsibility for deciding whether, while randomisation is in progress, the un-blinded results (or the un-blinded results for a particular subgroup), should be revealed to the TSC. They will do this if, and only if, two conditions are satisfied: (1) the results provide proof beyond reasonable doubt that treatment is on balance either definitely harmful or definitely favourable for all, or for a particular category of, participants in terms of the major outcome, and (2) the results, if revealed, would be expected to substantially change the prescribing patterns of clinicians who are already familiar with other trial results that exist. Exact criteria for ‘proof beyond reasonable doubt’ are not, and cannot be, specified by a purely mathematical stopping rule, but they are strongly influenced by such rules. This is in agreement with the Haybittle-Peto stopping rule [33, 34], whereby an interim analysis of major endpoint would generally need to involve a difference between treatment and control of at least three standard errors to justify premature disclosure. An interim subgroup analysis would have to be even more extreme to justify disclosure. This rule has the advantage that the exact number and timing of interim analyses need not be pre-specified. In summary, the stopping rules require extreme differences to justify premature disclosure and involve an appropriate combination of mathematical stopping rules and scientific judgment. To date, seven interim analyses by the DMC have been conducted with no recommendation for early stopping.

## Data management and analysis software

The clinical database management system for WOMAN Trial was built to comply with ICH-GCP and uses MySQL and its accompanying manuals. PHP was used to develop the dynamic web pages for user interface. The database was developed by Sealed Envelope Ltd. Data are collected at each participating site and transmitted directly to the clinical trials unit (CTU) via the database. Where there is poor internet connection, the paper CRFs can be sent by fax or via email. Data checks and cleaning are performed by the CTU. Data items to be coded including Adverse Event term and terms used to describe ‘other’ causes of death on the Outcome Form are coded using MedDRA Version 12 [27]. The final database lock will take place at the end of the trial within 3 months from the time when the ‘last patient’ in the ‘last follow-up’ has completed the trial. Data will be exported for statistical analysis using the most recent version of Stata (StataCorp LP, College Station, Texas, USA).

## Data sharing

Following publication of the primary and secondary analyses detailed in this statistical analysis plan, the trial data will be made available via our data sharing portal - The Free Bank of Injury and emergency Research Data (freeBIRD) website (http://freebird.Lshtm.ac.uk). This will allow for maximum utilisation of the data to improve patient care and advance medical knowledge.

## Discussion

This report presents and justifies the changes to our previously published protocol. The main changes are an increased sample size from 15,000 patients to 20,000 and changes to our planned subgroup analyses, time from delivery to randomisation is a new subgroup, and the removal of the subgroup ‘use of uterotonic prophylaxis’.

We have presented our plan for the statistical analyses in advance of the database lock and un-blinding to guard against data dependent analyses.

The WOMAN trial will provide reliable evidence for the efficacy of tranexamic acid in the prevention of death, hysterectomy and other outcomes important to patients and clinicians. Aditional file 4 lists the collaborating investigators who have participated in the WOMAN Trial to date.

## Additional files

Additional file 1: List of ethics approval for participating countries and sites. (DOCX 19 kb) Additional file 2: Primary outcome monitoring form. (DOCX 34 kb) Additional file 3: Guidance for diagnosis of complications. (DOCX 59 kb) Additional file 4: Trial collaborators. (DOCX 23 kb)

## Abbreviations

AE: adverse event
CI: confidence interval
CONSORT: Consolidated Standards of Reporting Trials
CRASH-2 trial: Clinical Randomisation of an Antifibrinolytic in Significant Haemorrhage Trial
CTU: clinical trials unit
DIC: disseminated intravascular coagulation
DMC: data monitoring committee
DVT: deep vein thrombosis
ICH-GCP: International Conference on Harmonisation of Technical Requirements for Registration of Pharmaceuticals for Human Use – Good Clinical Practice
ICU: intensive care unit
ITT: intention to treat
MySQL: My Structured Query Language
PAI: plasminogen activator inhibitors
PE: pulmonary embolism
PHP: PPH hypertext preprocessor
PPH: post-partum haemorrhage
SAE: serious adverse event
SUSAR: suspected unexpected serious adverse reaction
SD: standard deviation
TE: thromboembolic events
TPA: tissue plasminogen activator
TXA: tranexamic acid
UK: United Kingdom
RR: relative risk
VTE: venous thromboembolic event
WOMAN: World Maternal Antifibrinolytic Trial
