# Supplementary material for: The effect of tranexamic acid on the risk of death and hysterectomy in women with post-partum haemorrhage: statistical analysis plan for the WOMAN trial
Source: Trials. 2016 May 17;17:249. doi: 10.1186/s13063-016-1332-2 (PMC4869395; doi:10.1186/s13063-016-1332-2)
Supplement: Additional file 1: — List of ethics approval for participating countries and sites. (DOCX 19 kb) [file 13063_2016_1332_MOESM1_ESM.docx]

**WOMAN TRIAL ETHICS APPROVALS (Country, Site, Date granted, Reference number if exists)**

**Albania national approval Oct-09**

Lezha Regional Hospital Jul-12

Obstetric Gynaecology University Hospital K Gliozheni Dec-14

Regional Hospital Elbasan Oct-12

Regional Hospital Fier Dec-14

**Bangladesh national approval Jul-10, 04/2010-11**

Ad-din Women's Medical College & Hospital Jan-10

Chittagong Medical College Hospital Jun-12

Dhaka Medical College Hospital Jul-12, DMC-MEU/ECC/2014/17(D)

Ibn Sina Medical College Hospital Feb-13

Rajshahi Medical College Hospital Jun-10

**Burkina Faso national approval Apr-11, 2011-000051/MS/MRS/CERS**

Centre Hospitalier Regional de Dedougou Dec-10, 2010-10500/MS/SG/CHR.DDG/DG

Centre Hospitalier Universitaire Souro Sanou Nov-11, 2011-01174/MS/RHBS/DRS

**Cameroon national approval Feb-10, 045/CNE/SE/2010**

Banyo District Hospital Sep-11, 392/10/R/MSP/DRSPA/SSD/HD BYO

Centre Hospitalier et Universitaire Yaounde Jun-10, 120/CNE/SE/2010

Dschang District Hospital Sep-10

Hopital Laquintinie de Douala Apr-10, 1300/AUT/HLD/SCM; 2052/PR/MSP/HLD/SCM

Kumba District Referral Hospital Jun-10, 1075/DHK/M/06/2010

Regional Hospital Limbe Mar-10, A.1.5/HA/MPH/SWR/RHL/DO

Sa'a District Hospital Sep-10

St Theresa's Catholic Hospital Mar-10, 0075/10

Yaounde Central Hospital Nov-11, 297 HCY/cm

Yaounde Gynaeco-Obstetric and Paediatric Hospital Sep-10

**Colombia national approval Mar-11, 10020494**

Fundacion Valle del Lili Mar-10

**Democratic Republic of Congo national approval Feb-14**

Centre de Sante de Reference Albert Barthel Mar-14, 004/03/2014

Centre de Sante de Reference Kahembe Apr-14, 017/CSR/KAH/201Y

Centre Hospitalier Notre Dame d'Afrique Apr-14

Centre Medical ADEBECO Mar-14, Abedeco/CODI/2014

Centre Medical VUHE May-14

Centre de Sante de Reference Carmel Mar-14, 0045/csr Carmel/CEI/2014

Groupe d'entraide et de Solidarite Medicale Feb-14

Hope Medical Center Feb-14, 001/DM/AE/A.01/2014

Provincial Hospital Goma Mar-14

Virunga General Hospital Mar-14, 56/HGR/CBCA/VIR-GOM/MD/014

**Egypt national approval Aug-10, HM000046**

Mataria Teaching Hospital Nov-10

**Ethiopia national approval Mar-11, 310/162/03**

Jimma University Hospital Dec-14, RPGC/555/2014

St. Paul's Hospital Millennium Medical College Feb-15, P.M/23/8/

**Ghana national approval Feb-11, GHS-ERC:01/7/10**

Ashanti Mampong Municipal Hospital Aug-10, CHRPE/106&117/09

Komfo Anokye Teaching Hospital Aug-10, CHRPE/117/

**Jamaica national approval Jun-10, LC 01/09/2010**

University Hospital of the West Indies Dec-09

**Kenya national approval Jan-15, KNH-ERC/MOD/404**

AIC Kijabe Hospital Sep-14

Bungoma District Hospital Jan-14

Coast Provincial General Hospital May-11, ADM/1/29/vol.1

Garissa Provincial General Hospital Feb-15, No P/NO999047950/(7)

Kenyatta National Hospital Jun-11, KNH/OBS/GYN/16

Moi Teaching and Referral Hospital Mar-15, IREC/2010/59

Mwingi District Hospital Jul-14, MEDSUPT/HAO/CORR/VOL.1/105

Nakuru Provincial General Hospital Apr-15, RII/VOL.I/08

The Nairobi Hospital Feb-14, TNH/ADMIN/CEO/12/02/14

**Nepal national approval Jun-10, 1384**

Birat Hospital and Research Centre Oct-11

BP Koirala Institute of Health Sciences Jan-10, ACD.317/D6

Mid Western Regional Hospital Jul-11

Nepal Medical College Teaching Hospital Jan-10

**Nigeria national approval Oct-09, NHREC/01/01/2007-22/09/2011**

Abubakar Tafawa Balewa University Teaching Hospital Jun-14, ATBUTH/ADM/42/Vol I

Adeoyo Maternity Hospital Feb-10, AD 13/479/

Ahmadu Bello University Teaching Hospital Sep-10, ABUTH/PGO/COMM9

Ajeromi General Hospital Jun-14, LREC/09/11

Aminu Kano Teaching Hospital Jun-13, AKTH/MAC/SUB/12A/P-3/VI/1267

Braithwaite Memorial Specialist Hospital Feb-11, UNTH/CSA 3

Delta State University Teaching Hospital Mar-10, CHW/E/140

Ekiti State University Teaching Hospital Feb-11, UI/EC/09/0

Federal Medical Centre Abeokuta Mar-10, FMCA/470/HREC/2014

Federal Medical Centre Azare Nov-09, FMCA/DOM/3

Federal Medical Centre Vida Feb-11, HREC/PR/10

Federal Medical Centre Birnin-Kebbi Dec-09

Federal Medical Centre Gusau Nov-09, FMCGS/01/0

Federal Medical Centre Ido-Ekiti Mar-10, ERC/2009/1

Federal Medical Centre Katsina Nov-09

Federal Medical Centre Lokoja Feb-10

Federal Medical Centre Makurdi Jul-09, FMH/FMC/ME

Federal Medical Centre Owerri Mar-10, FMC/OW/HCS

Federal Medical Centre Owo Mar-10

Federal Medical Centre Umuahia Dec-13, UNTH/CSA 3

Federal Medical Centre Yenagoa Dec-13, UNTH/CSA 3

Federal Teaching Hospital Abakaliki Jun-14, UNTH/CSA 3

General Hospital Minna Sep-13, HREC/PR/10

Gwarimpa General Hospital Dec-11, FHREC/2010

Irrua Specialist Teaching Hospital Aug-09, ISTHREC09/

Jos University Teaching Hospital Jul-10, JUTH/DCS/A

Karshi General Hospital Feb-15, FHREC/2010/01/3/24-02-10

Kogi State Specialist Hospital Apr-10, KSSH/ETH/C

Ladoke Akintola University of Technology Teaching Hospital Nov-09, LTH/EC/200

Lagos Island Maternity Hospital Jan-11

Lagos State University Teaching Hospital Feb-10, LREC/09/11

Lagos University Teaching Hospital Dec-09, ADM/DCST/2

Maitama District Hospital Mar-10, FHREC/2010

Mother & Child Hospital Akure Aug-10

National Hospital Abuja Mar-10, NHA/ADMIN/

Nnamdi Azikiwe University Teaching Hospital Oct-09, NAUTH/CS/6

Nyanya General Hospital May-12, FHREC/2010

Obafemi Awolowo University Teaching Hospital Feb-10, ERC/2010/0

Plateau State Specialist Hospital Jul-10

Seventh Day Adventist Hospital Nov-09

State Specialist Hospital Akure Aug-14, UI/EC/09/0

University College Hospital Ibadan Dec-09, UI/EC/09/0

University of Abuja Teaching Hospital Dec-09, HREC/PR/10

University of Calabar Teaching Hospital Apr-10

University of Ilorin Teaching Hospital Mar-10, UITH/CAT/1

University of Maiduguri Teaching Hospital Feb-10

University of Nigeria Teaching Hospital Enugu Feb-14, UNTH/CSA 3

University of Uyo Teaching hospital Aug-09, UUTH/AD/S/

Usmanu Danfodiyo University Teaching Hospital Feb-14, UDUTH/HREC/2015/No.326

Wesley Guild Hospital Sep-12, ERC/2010/0

**Pakistan national approval Oct-14, 4-87/11/NBC-64/RDC/35**

Ayub Teaching Hospital Sep-13

Bolan Medical Complex Hospital May-12, EC 01-4/2012

CGH Cantonment General Hospital Nov-12

Combined Military Hospital Kharian Dec-14

Combined Military Hospital Lahore Apr-13

Dera Ismail Khan District Teaching Hospital Apr-14, /293/GMC

Fatima Bai Hospital Oct-13

Fatima Memorial Hospital May-12

Federal Government Poly Clinic Hospital Islamabad Dec-14, FGPC 1/4306/2012

Holy Family Hospital Jan-15

Isra University Hospital Dec-14

Jinnah Hospital Lahore Sep-13, 1425/ERB/14th

Kahota Research Laboratory General Hospital Jul-14, ERC-14-07-01

Lady Aitchison Hospital Apr-14, 687/RC/KEMU

Lady Reading Hospital Jul-12, 161/IREB/PGMI

Liaquat Memorial Women & Children Hospital Kohat Jan-14, 32/KIMS/IRBB/RP/2014

Liaquat National Hospital Jul-11, 0032-2011 LNH-ERC

Liaquat University Hospital Jan-15, LUMHS/REC/=232

MCH Centre PIMS Sep-14

Mian Mohammad Trust Hospital Feb-11

Military Hospital Rawalpindi May-15

Nescom Hospital Nov-13

Nishtar Hospital Dec-12, 2248/88/NMC&H

Pakistan Railway Hospital Jan-15, Riphah/IIMC-REC/15-88

Patel Hospital Jan-15

People's University of Medical and Health Sciences Nov-13, PUMHS/SBA/VC/PA:/1372

Punjab Medical College Oct-12, F.No.48-ERC/2012-13/PMRC/PMC/120

Rehman Medical Institute Private Limited Sep-14, RMI/GYN/WOMAN/01

Services Hospital Lahore Nov-13, 44/IRB/SIMS

Shalamar Hospital Jan-15, SMDC/Ortho/15-01/295

Sharif Medical & Dental City Nov-12, SMDC/Obs & Gynae/ /2012

Shifa International Hospital Aug-14, IRB # 2027-076-2012

Sir Ganga Ram Hospital Lahore Feb-13, 030 JFJMC

Sobhraj Maternity Hospital KMC Sep-13, 0026/13

Zainab Panjwani Memorial Hospital Dec-14

Ziauddin University Hospital Jan-13, 0591112HSGYN

**Papua New Guinea national approval May-15**

Port Moresby General Hospital Apr-15

**Sudan national approval Oct-10, Committee 26/2010**

Elmek Nimir University Hospital Sep-13

El-Obeid Teaching Hospital Oct-12

Gadarif Obstetrics and Gynaecology Hospital Sep-13

Kassala New Hospital Al Saudi Oct-12

Khartoum North Teaching Hospital Mar-12

Khartoum Teaching Hospital Oct-10

Kosti Hospital Aug-14

Omdurman Maternity Hospital Oct-10

Soba University Hospital Oct-10

Wad Medani Teaching Hospital of Obstetrics and Gynaecology Sep-13

**Tanzania national approval Sep-10, NIMR/HQ/R.8c/Vol.II/174**

Bugando Medical Centre Sep-10

Hospitali Teule Muheza Designated District Hospital Dec-09

Muhimbili National Hospital Jun-10

Mwananyamala Municipal Hospital Mar-11, MH/RES/1/5

Sekou Toure Regional Hospital Nov-10

Temeke Municipal Hospital May-15

**Uganda national approval Nov-14, HS 1073**

Adjumani Hospital Aug-11, HOSP/PER/154/4

Angal St Luke Hospital Aug-12

Church of Uganda Kisiizi Hospital Oct-09

Entebbe General Hospital Oct-12

Mbarara Hospital Feb-14, DMS 6

Mubende Regional Referral Hospital Oct-12

Mulago Hospital Jul-11

Nyakibale Hospital Aug-11

St Francis Hospital Buluba Oct-14

Uganda Martyrs Ibanda Hospital Apr-10

**United Kingdom national approval Feb-11, 10/H0505/111 covering:**

City Hospital Nottingham

Liverpool Women's NHS Foundation Trust

Queen's Medical Centre, Nottingham University Hospitals Trust

St Mary's Hospital Manchester

St Thomas' Hospital, Guy's and St Thomas' NHS Foundation Trust

Sunderland Royal Hospital, City Hospitals Sunderland NHS Trust

The Royal Victoria Infirmary, Newcastle Upon Tyne Hospitals NHS Trust

**Zambia national approval Aug-14, 009-02-10**

Chipata General Hospital Jul-10

Kafue District Hospital Jul-13

Livingstone General Hospital Sep-14

St Francis Hospital Katete Oct-14
