# Supplementary material for: The effect of tranexamic acid on the risk of death and hysterectomy in women with post-partum haemorrhage: statistical analysis plan for the WOMAN trial
Source: Trials. 2016 May 17;17:249. doi: 10.1186/s13063-016-1332-2 (PMC4869395; doi:10.1186/s13063-016-1332-2)
Supplement: Additional file 2: — Primary outcome monitoring form. (DOCX 34 kb) [file 13063_2016_1332_MOESM2_ESM.docx]

| 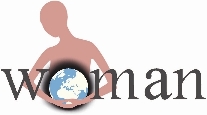 | **PRIMARY OUTCOME MONITORING** | | | | **Site Name:** |  |
| --- | --- | --- | --- | --- | --- | --- |
| **Randomisation number (box/pack**) | |  | | | | |
| (1) Date of delivery | |  | | (2) Time of delivery |  | |
| (3) Date of randomisation | |  | | (4a) Time of randomisation |  | |
|  |  |  |  | (4b) Time of administration of trial treatment |  | |
| (5a) Was the decision to do a hysterectomy made before delivery or the onset of PPH? | | YES/NO | | (5b) How evidenced? | | |
| (6a) Was the decision to do a hysterectomy made before randomisation? | | YES/NO | | (6b) How evidenced? | | |
| (7) Please circle the main reason for doing the hysterectomy | | 1. To remove an abnormally adherent placenta (previa or accreta)  2. To remove a severely ruptured, damaged or infected uterus  3. To remove another uterine abnormality – e.g. uterinemyoma  4. To control bleeding  5. Other reason – describe:____________________________________________ | | | | |
| (8) Date of hysterectomy | |  | | (9) Time of hysterectomy |  | |
| (10) Primary cause of death | |  | | | | |
| (11) Date of death | |  | (12) Time of death | |  | |
| **ANY ACTION REQUIRED (Please attach an additional sheet if space is insufficient)** | | | | | | |
| Monitor Signature: Date: | | | | | | |
| **ANY DATA TO BE CHANGED – PLEASE DETAIL BELOW (to be signed by Site Investigator)** | | | | | | |
| Site Investigator Signature: Date: | | | | | | |
| **SOURCE DATA VERIFIED BY** | | | | | | |
| **NAME: SIGNATURE: DATE:** | | | | | | |
| **CTU ACTION TAKEN** | | | | | | |
| **Date query raised : Date database updated:** | | | | | | |
| **Any other action:** | | | | | | |
| **NAME (and position):** | | | | | | |
| **SIGNATURE: DATE:** | | | | | | |
