# Supplementary material for: The effect of tranexamic acid on the risk of death and hysterectomy in women with post-partum haemorrhage: statistical analysis plan for the WOMAN trial
Source: Trials. 2016 May 17;17:249. doi: 10.1186/s13063-016-1332-2 (PMC4869395; doi:10.1186/s13063-016-1332-2)
Supplement: Additional file 3: — Guidance for diagnosis of complications. (DOCX 59 kb) [file 13063_2016_1332_MOESM3_ESM.docx]

GUIDANCE FOR DATA COLLECTION


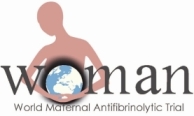


Outcome form Section 5

**Introduction**

This guidance is to allow standardised reporting of complications in the WOMAN trial. Before circling ‘YES’ to questions in Section 5 of the OUTCOME FORM, please ensure that the event fulfils the definition given below.

**5. COMPLICATIONS** *(circle one option on every line)*

| **a)** Pulmonary embolism | YES | NO |
| --- | --- | --- |
| **b)** Deep vein thrombosis | YES | NO |
| **c)** Stroke | YES | NO |
| **d)** Myocardial infarction | YES | NO |
| **e)** Renal failure | YES | NO |
| **f)** Cardiac Failure | YES | NO |
| **g)** Respiratory Failure | YES | NO |
| **h)** Hepatic Failure | YES | NO |
| **i)** Sepsis | YES | NO |
| **j)** Seizures | YES | NO |

**PLEASE NOTE:** Any complications not listed above should be reported as per protocol using an Adverse Event Reporting form.

1. **Pulmonary embolism (PE)**

The diagnosis of PE requires one of the following:

- High probability ventilation/perfusion lung scan
- Intraluminal filling defect of segmental or larger artery on Helical CT scan
- Intraluminal filling defect on a pulmonary angiography

**OR**

- A positive diagnostic test for DVT + low or intermediate probability ventilation/perfusion lung scan or a subsegmental defect on a helical CT scan

1. **Deep vein thrombosis (DVT)**

The diagnosis of DVT requires both clinical assessment and confirmation by one of the following tests:

- A persistent intraluminal filling defect on contrast venography
- Non-compressibility of one or more venous segments on B mode compression ultrasonography
- Intraluminal filling defect on a contrast CT scan

1. **Stroke**

This is defined as ‘a new focal neurological deficit with signs and symptoms lasting more than 24 hours’[^1^](#_ENREF_1)

1. **Myocardial infarction (MI)**

The universal definition of myocardial infarction[^2^](#_ENREF_2) should be used: Detection of rise and/or fall of cardiac biomarker values (preferably troponin) with at least one value above the 99th percentile of the upper reference limit and with at least one of the following:

- Symptoms of ischaemia
- ECG abnormalities: new or presumably new significant ST-T changes or new LBBB (left bundle branch block) or pathological Q waves
- Imaging evidence of new loss of viable myocardium, or new regional wall motion abnormality
- Identification of an intracoronary thrombus by angiography or autopsy
- Cardiac death with symptoms suggestive of myocardial ischaemia
- Stent thrombosis associated with MI when detected by coronary angiography or autopsy in the setting of myocardial ischaemia and with a rise and/or fall of cardiac biomarker values with at least one value above the 99^th^ percentile URL

1. **Renal failure**

Renal failure is defined when one of the following criteria is met in adult population:

- Serum creatinine rise ≥ 26µmol/L (0.29 mg/dl) within 48 hours or a 50% or greater rise in serum creatinine known or presumed to have occurred within the past 7 days.[^3^](#_ENREF_5)
- Urine output is < 0.5ml/kg/hr for >6 consecutive hour.[^3^](#_ENREF_5)
- In those with pre-existing renal disease - a serum creatinine rise of 200% or more from index serum creatinine or serum creatinine increased to 350 μmol/L (4mg/dl).[^4^](#_ENREF_6)

1. **Cardiac failure**

According to ESC Guidelines for the diagnosis and treatment of acute and chronic heart failure 2012, the diagnosis of heart failure requires three conditions to be satisfied^5^:

1. Presence of symptoms typical of heart failure (i.e. Breathlessness, orthopnoea, paroxysmal nocturnal dyspnoea, reduced exercise tolerance, fatigue, tiredness)
2. Presence of signs typical of heart failure (i.e. elevated jugular venous pressure, hepatojugular reflux, third heart sound or gallop rhythm, increased time to recover after exercise)
3. Presence of reduced left ventricular ejection fraction or relevant structural heart disease (LV hypertrophy/LA enlargement) and/or diastolic dysfunction.
4. **Respiratory failure**

Respiratory failure is diagnosed when arterial blood gas shows PaO2 of <60 mmHg (8.0 kPa) on room air at sea level.[^6^](#_ENREF_4)

1. **Hepatic failure**

Liver failure is diagnosed when deterioration in liver function is accompanied by changes in mental status and coagulopathy.

1. **Sepsis**

The diagnosis of sepsis is based on the presence of **both**:

- Infection and
- A systemic inflammatory response syndrome (SIRS).[^7^](#_ENREF_3) SIRS requires two or more of the following:
  - Temperature <36 °C or >38 °C
  - [Heart rate](http://en.wikipedia.org/wiki/Heart_rate) >90 beats/min
  - Respiratory rate >20 breaths/min
  - White blood cell count <4x109/L (<4000/mm³) or >12x109/L (>12,000/mm³)

1. **Seizures**

This is based on a clinical judgement.^8^

**References**

1. Sung V, Johnson N, Granstaff U, et al. Sensitivity and specificity of stroke symptom questions to detect stroke or transient ischemic attack*.* *Neuroepidemiology,* 2011. 36(2):100-4.
2. Steg P, James S, Atar D, et al. ESC Guidelines for the management of acute myocardial infarction in patients presenting with ST-segment elevation*.* *Eur. Heart J.,* 2012. 33(20):2569-619.
3. NICE guidelines 169 – prevention, detection and management of acute kidney injury*.*
4. Zhou Q, Zhao C, Xie D, et al. Acute and acute-on-chronic kidney injury of patients with decompensated heart failure: Impact on outcomes*.* *BMC Nephrol.,* 2012. 13(1).
5. McMurray J. J. V et al. ESC Guidelines for the diagnosis and treatment of acute and chronic heart failure 2012. E*uropean Heart Journal* (2012) 33, 1787–1847
6. Hart N. Respiratory failure*.* *Medicine,* 2008. 36(5):242-5.
7. Bone R, Balk R, Cerra F, et al. Definitions for sepsis and organ failure and guidelines for the use of innovative therapies in sepsis*.* *Chest,* 1992. 101(6):1644-55.
8. Cuthill FM and Espie CA. Sensitivity and specificity of procedures for the differential diagnosis of epileptic and non-epileptic seizures: a systematic review*.* *Seizure,* 2005. 14(5):293-303.
