# Supplementary material for: The effect of tranexamic acid on the risk of death and hysterectomy in women with post-partum haemorrhage: statistical analysis plan for the WOMAN trial
Source: Trials. 2016 May 17;17:249. doi: 10.1186/s13063-016-1332-2 (PMC4869395; doi:10.1186/s13063-016-1332-2)
Supplement: Additional file 4: — Trial collaborators. (DOCX 23 kb) [file 13063_2016_1332_MOESM4_ESM.docx]

**TRIAL COLLABORATORS** *(Country: Site, Principal Investigator)*

*Albania:* Lezha Regional Hospital, Leon Kaza; Obstetric Gynaecology University Hospital K Gliozheni, Kastriot Dallaku; Regional Hospital Elbasan, Armida Tola; Regional Hospital Fier, Besnik Brahimaj.

*Bangladesh:* Ad-din Women's Medical College & Hospital, Sayeba Akhter; Chittagong Medical College Hospital, Rokeya Begum; Dhaka Medical College Hospital, Ferdousi Islam;

Ibn Sina Medical College Hospital, Nazlima Nargis;

Rajshahi Medical College Hospital, Shahela Jesmin.

*Burkina Faso:* Centre Hospitalier Regional de Dedougou, Roamba Pabakba; Centre Hospitalier Universitaire Souro Sanou, Moussa Bambara.

*Cameroon:* Banyo District Hospital, Eta Ngole Mbong; Centre Hospitalier et Universitaire Yaounde, Pierre Marie Tebeu; Dschang District Hospital, Kenfack Bruno; Hopital Laquintinie de Douala, Charlotte Tchente Nguefack; Kumba District Referral Hospital, Etienne Asonganyi; Regional Hospital Limbe, Andre Gaetan Simo Wambo; Sa'a District Hospital, Georges Nko'ayissi; St Theresa's Catholic Hospital, Mutsu Bumaha Venantius; Yaounde Central Hospital, Jeanne Fouedjio; Yaounde Gynaeco-Obstetric and Paediatric Hospital, Lilian N Ngwana Banmi.

*Colombia:* Fundacion Valle del Lili, Maria Fernanda Escobar.

*Democratic Republic of Congo:* Centre de Sante de Reference Albert Barthel, Badibanga Musau; Centre de Sante de Reference Kahembe, Nzanzu Kikuhe Jason; Centre Hospitalier Notre Dame d'Afrique, Jean Robert Lubamba; Centre Medical ADEBECO, Roger Paluku; Centre Medical VUHE, Herman Kalyana; Centre de Sante de Reference Carmel, Kato Faida; Groupe d'entraide et de Solidarite Medicale, Serge Mulyumba; Hope Medical Center, Mateus Kambale Sahani; Provincial Hospital Goma, Willis Missumba; Virunga General Hospital, Phanny Kambere Simisi.

*Egypt:* Mataria Teaching Hospital, Magdy Abd El Rahman.

*Ethiopia:* Jimma University Hospital, Hailemariam Segni; St Paul's Hospital Millennium Medical College, Abdulfetah Abdulkadir Abdosh.

*Ghana:* Ashanti Mampong Municipal Hospital, Kwadwo A Nyarko-Jectey; Komfo Anokye Teaching Hospital, Henry Sakyi Opare-Addo.

*Jamaica:* University Hospital of the West Indies, Horace Melford Fletcher.

*Kenya:* AIC Kijabe Hospital, Brian Liyayi; Bungoma District Hospital, Kubasu Wekesa; Coast Provincial General Hospital, Faiza A Nassir; Garissa Provincial General Hospital, Vincent Oyiengo; Kenyatta National Hospital, Zahida Qureshi; Moi Teaching and Referral Hospital, Hillary Mabeya; Mwingi District Hospital, Bonface Nzioki Musila; Nakuru Provincial General Hospital, Amos Otara; The Nairobi Hospital, Alex Nyakundi Bosire.

*Nepal:* Birat Hospital and Research Centre, Gyanendra Man Singh Karki; BP Koirala Institute of Health Sciences, Mohan C Regmi; Mid-Western Regional Hospital, Bhola Ram Shrestha; Nepal Medical College Teaching Hospital, Pramila Pradhan.

*Nigeria:* Abubakar Tafawa Balewa University Teaching Hospital, Lamaran A Dattijo; Adeoyo Maternity Hospital, Akintunde Kehinde Ayinde; Ahmadu Bello University Teaching Hospital, Sulayman Hajaratu Umar; Ajeromi General Hospital, Nurudeen Onaolapo; Aminu Kano Teaching Hospital, Hadiza Galadanci; Braithwaite Memorial Specialist Hospital, Paul Ledee Kua; Delta State University Teaching Hospital, Lawrence Omo-Aghoja; Ekiti State University Teaching Hospital, JO Awoleke; Federal Medical Centre Abeokuta, Moses Oluwafemi Alao; Federal Medical Centre Azare, Umar Ibrahim; Federal Medical Centre Bida, Ikemefuna Nwosu; Federal Medical Centre Birnin-Kebbi, Yusuf Tanko Sununu; Federal Medical Centre Gusau, Kamil Shoretire; Federal Medical Centre Ido-Ekiti, Augustine Adebayo Adeniyi; Federal Medical Centre Katsina, Ibrahim Habib; Federal Medical Centre Lokoja, Onile Temitope; Federal Medical Centre Makurdi, Andrea Jogo; Federal Medical Centre Owerri, Emily Nzeribe; Federal Medical Centre Owo, Olufemi Akinsanya; Federal Medical Centre Umuahia, Enyinnaya Chikwendu Nduka; Federal Medical Centre Yenagoa, Amaitari C Bariweni; Federal Teaching Hospital Abakaliki, OUJ Umeora; General Hospital Minna, Mohammed Gana; Gwarimpa General Hospital, Osagie Osayande; Irrua Specialist Teaching Hospital, Felix Okogbo; Jos University Teaching Hospital, Josiah Mutihir; Karshi General Hospital, Ola Nene Okike; Kogi State Specialist Hospital, Osawaru Godwin Ukpomwan; Ladoke Akintola University of Technology Teaching Hospital Ogbomoso, Adetunji Oladeni Adeniji; Ladoke Akintola University of Technology Teaching Hospital Osogbo, Adeniyi Fasanu; Lagos Island Maternity Hospital, Simeon Bazuaye; Lagos State University Teaching Hospital, Oluwarotimi Ireti Akinola; Lagos University Teaching Hospital, Omololu Adegbola; Maitama District Hospital, Frank Alu; Mother & Child Hospital Akure, Adesina L Akintan; National Hospital Abuja, Olubunmi Ladipo; Nnamdi Azikiwe University Teaching Hospital, Joseph Ikechebelu; Nyanya General Hospital, Hadiza Abdulaziz Idris; Obafemi Awolowo University Teaching Hospital, Babalola Adeyemi; Plateau State Specialist Hospital, William Nengak Golit; Seventh Day Adventist Hospital, Owigho Peter Opreh; State Specialist Hospital Akure, Theresa Azonima Irinyenikan; University College Hospital Ibadan, Oladapo Olayemi; University of Abuja Teaching Hospital, Olatunde Onafowokan; University of Calabar Teaching Hospital, Saturday Job Etuk; University of Ilorin Teaching Hospital, Abiodun Peter Aboyeji; University of Maiduguri Teaching Hospital, Audu Idrisa; University of Nigeria Teaching Hospital Enugu, Eziamaka Pauline Ezenkwele; University of Uyo Teaching hospital, Aniefiok Umoiyoho; Usmanu Danfodiyo University Teaching Hospital, Swati Singh; Wesley Guild Hospital, Babalola Adeyemi.

*Pakistan:* Ayub Teaching Hospital, Shehla Noor; Bolan Medical Complex Hospital Unit IV, Tasneem Ashraf; CGH Cantonment General Hospital, Nighat Shaheen; Combined Military Hospital Kharian, Umbreen Akram; Combined Military Hospital Lahore, Nilofar Mustafa; Dera Ismail Khan District Teaching Hospital, Naseem Saba; Fatima Bai Hospital, Shabeen Naz Masood; Fatima Memorial Hospital, Shamayela Hanif; Federal Government Poly Clinic Hospital, Naila Israr; Holy Family Hospital Gyn & Obs Unit I, Rizwana Chaudhri; Holy Family Hospital, Gyn & Obs Unit II, Fehmida Shaheen; Isra University Hospital, Pushpa Sirichand Sachdev; Jinnah Hospital Lahore, Amtullah Zarreen Khan; Kahota Research Laboratory General Hospital, Tahira Batool; Lady Aitchison Hospital, Abida Sajid; Lady Reading Hospital, Sadaqat Jabeen; Liaquat Memorial Women & Children Hospital, Fouzia Gul; Liaquat National Hospital, Haleema Akhtar Hashmi; Liaquat University Hospital LUMHS Gynae Unit I, Roshan Ara Qazi; MCH Centre PIMS, Obs and Gynae Unit I , Syeda Batool Mazhar; MCH Centre PIMS, Obs and Gynae Unit II, Nasira Tasnim; Mian Mohammad Trust Hospital, Ambreen Ikram; Military Hospital Rawalpindi, Naila Tahir Kyani; Nescom Hospital, Nabila Salman;

Nishtar Hospital Unit I, Hajira Masood; Nishtar Hospital Unit II, Huma Quddusi; Nishtar Hospital Unit III, Shahid Irshad Rao; Pakistan Railway Hospital, Fareesa Waqar; Patel Hospital, Samina Saleem Dojki; People's University of Medical and Health Sciences, Razia Bahadur Khero; Punjab Medical College, Samina Kausar; Rehman Medical Institute Private Limited, Saeeda Majeed; Services Hospital Lahore Unit II, Rubina Sohail; Services Hospital Lahore Unit III, Tayyiba Wasim; Shalamar Hospital, Lubna Riaz Dar; Sharif Medical & Dental City, Maimoona Hafeez; Shifa International Hospital, Nabia Tariq; Sir Ganga Ram Hospital Lahore, Shamsa Humayun; Sobhraj Maternity Hospital KMC, Samina Iqbal; Zainab Panjwani Memorial Hospital, Samar Amin; Ziauddin University Hospital Clifton Campus, Rubina Hussain; Ziauddin University Hospital Nazimabad Campus, Shama Chaudhry; Ziauddin University Hospital North Nazimabad Campus, Shazia Sultana; Ziauddin University Kemari Campus, Habiba Sharaf Ali.

*Papua New Guinea:* Port Moresby General Hospital, Glen Mola,

*Sudan:* Elmek Nimir University Hospital, Saeed Abdelrahman Abdelgabar; El-Obeid Teaching Hospital, Khidir Awadalla; Gadarif Obstetrics and Gynaecology Hospital, Huaida Mardi Mohammed Ahmed; Kassala New Hospital Al Saudi, Abdalla Ali Mohammed; Khartoum North Teaching Hospital, Wisal Omer Mohammed Nabag; Khartoum Teaching Hospital, Duria Rayis; Kosti Hospital, Mahdi Bushra; Omdurman Maternity Hospital, Taha Umbeli Ahmed; Soba University Hospital, Hala Abdullahi; Wad Medani Teaching Hospital of Obstetrics and Gynaecology, Somia Ahmed.

*Tanzania:* Bugando Medical Centre, Anthony Massinde; Hospitali Teule Muheza Designated District Hospital, George Mtove; Muhimbili National Hospital, Hussein Kidanto; Mwananyamala Municipal Hospital, Sebastian Kitengile Ganyaka; Sekou Toure Regional Hospital, Rwakyendela Onesmo; Temeke Municipal Hospital, Muzdalifat Salim Abeid.

*Uganda:* Adjumani Hospital, Dominic Drametu; Angal St Luke Hospital, Grace Meregurwa; Church of Uganda Kisiizi Hospital, Francis Banya Ogwang; Entebbe General Hospital, Ayiko Ben Jackson; Mbarara Hospital, Joseph Ngonzi; Mubende Regional Referral Hospital, Patrick Komagum; Mulago Hospital, Christine Biryabarema; Nyakibale Hospital, Lema Felix; St Francis Hospital Buluba, Elizabeth Nionzima; Uganda Martyrs Ibanda Hospital, Emmanuel Byaruhanga.

*United Kingdom:* City Hospital Nottingham, Jim Thornton; Liverpool Women's NHS Foundation Trust, Zarko Alfirevic; Queen's Medical Centre, Nottingham University Hospitals Trust, Jim Thornton; St Mary's Hospital Manchester, Clare Tower; St Thomas' Hospital, Guy's and St Thomas' NHS Foundation Trust, Eugene Oteng-Ntim; Sunderland Royal Hospital, City Hospitals Sunderland NHS Trust, Kim Hinshaw; The Royal Victoria Infirmary, Newcastle Upon Tyne Hospitals NHS Trust, Paul Ayuk.

*Zambia:* Chipata General Hospital, Mathew Ng'ambi; Kafue District Hospital, Mwansa Ketty Lubeya; Livingstone General Hospital, Isaiah Hansingo; St Francis Hospital Katete, Ziche H Makukula; University Teaching Hospital Lusaka, Bellington Vwalika.

**TRIAL STEERING COMMITTEE:**

Professor Adrian Grant (previous Chair), Professor Sabaratnam Arulkumaran (Chair), Dr Kaosar Afsana, Dr Metin Gülmezoglu, Professor Beverley Hunt, Dr Oladapo Olayemi, Professor Ian Roberts, Ms Haleema Shakur.

**DATA MONITORING COMMITTEE:**

Professor Iain Chalmers (Chair), Dr Pisake Lumbiganon, Dr Gilda Piaggio.

**INTERNATIONAL ADVISORY COMMITTEE:**

Kastriot Dallaku (Albania), Sayeba Akter (Bangladesh), Nicolas Meda (Burkina Faso), Robert Tchounzou (Cameroon), Liliana Vallecilla (Colombia), Abdulfetah Abdulkadir Abdosh (Ethiopia), Zahida Qureshi (Kenya), Mohan Regmi (Nepal), Bukola Fawole (Nigeria), Rizwana Chaudhri (Pakistan), Mohamed Ahmed El Sheikh (Sudan), Hussein Kidanto (Tanzania), Christine Biryabarema (Uganda), Bellington Vwalika (Zambia).

**TRIAL COORDINATION:**

***Global Coordinating Centre***: Ian Roberts (Chief Investigator), Haleema Shakur (Project Director), Phil Edwards (Statistician), Monica Arribas (Assistant trial manager), Eni Balogun (Trial manager), Lin Barnetson (Data Manager), Collette Barrow (Trial administrator), Danielle Beaumont (Trial manager), Myriam Benyahia (Trial assistant), Lisa Cook (Assistant Trial Manager), Lauren Frimley (Clinical trials associate), Dan Gilbert (Data Assistant), Catherine Gilliam (Trial administrator), Nayia Golfi (Trial manager), Daniel Hetherington (Trial assistant), Robert Jackson (Data manager), Taemi Kawahara (Senior Trial manager), Katharine Ker (Lecturer), Sergey Kostrov (Systems Officer), Hakim Miah (Systems Manager), Aroudra Outtandy (Trial assistant), Nigel Quashie (Data manager), Anna Quinn (Data assistant), Tracey Pepple (Data Manager), Danielle Prowse (Data Assistant), Maria Ramos (Senior project administrator), Chris Roukas (Trial administrator), Chris Rubery (Data Assistant), Chelci Squires (Trial Assistant), Jemma Tanner (Trial assistant), Elizabeth Woods (Assistant trial manager).

***Nigeria National Coordinating Centre:*** Bukola Fawole (National Coordinator) Olusade Adetayo (Assistant trial coordinator), Olujide A Okunade (Assistant trial coordinator).

***Pakistan National Coordinating Centre:*** Rizwana Chaudhri (National Coordinator), Aasia Kayani (Research Coordinator), Kiran Javaid (Assistant Research Coordinator)
